# First person – Marinus Thein

**DOI:** 10.1242/bio.062378

**Published:** 2025-12-16

## Abstract

First Person is a series of interviews with the first authors of a selection of papers published in Biology Open, helping researchers promote themselves alongside their papers. Marinus Thein is first author on ‘ Effects of microtubule (de)tyrosination on the morphology and motility of *Trypanosoma brucei* and cross-talk with polyglutamylation’, published in BiO. Marinus is a PhD student in the lab of Professor Dr Klaus Ersfeld at University of Bayreuth, Bayreuth, Germany, investigating how the highly stable microtubule cytoskeleton of the parasite *Trypanosoma brucei* is regulated and how it achieves is structural integrity.


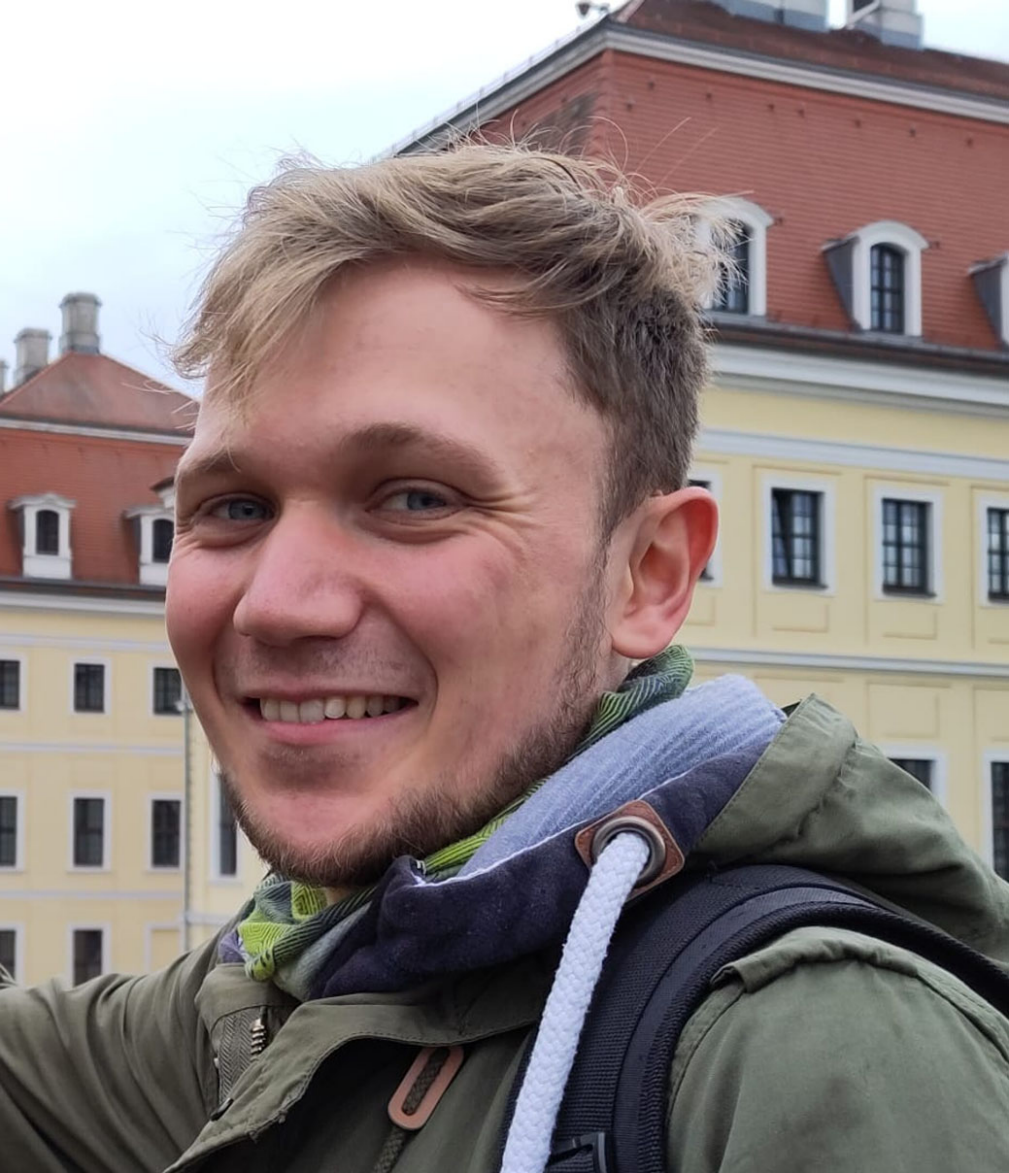


**Marinus Thein**

**Describe your scientific journey and your current research focus**

I have always been very interested in the natural sciences, especially biology and chemistry at school. So, I decided to combine them both and study biochemistry for my Bachelor’s and Master’s degrees. I joined the lab of Klaus Ersfeld for my Master's and stayed for my PhD. Here, we are interested in the dynamics of the microtubules forming the cytoskeleton of the parasite *Trypanosoma brucei*.

**Who or what inspired you to become a scientist?**

I don't think there is one singular person who inspired me. It is more science and life itself. I am always fascinated by how everything functions at molecular level, and investigating even the smallest parts of a whole complex mechanism/ organism can be so fascinating and eye-opening.

**How would you explain the main finding of your paper?**

Microtubules are essential cellular structures, and their malformation is often correlated with neurological diseases. This work further explores our understanding of how different sets of modifications on the essential microtubule proteins correlate with and regulate each other.

**What are the potential implications of this finding for your field of research?**

This work demonstrates that the various microtubule modifications do not occur in isolation, but, rather, they are interconnected and their formation is dependent on each other.

**Figure:**
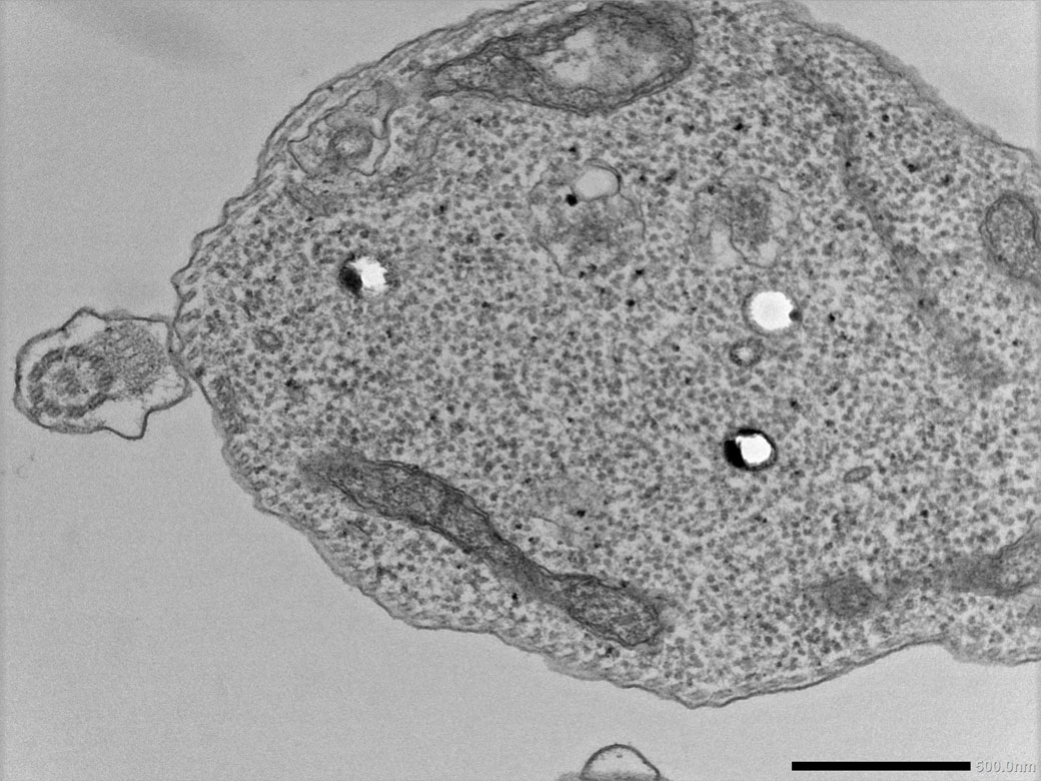
Transmission electron microscopy image of a cross-section of a trypanosome.

**Which part of this research project was the most rewarding?**

Probably noticing this cross-talk between the tubulin modifications directly in the cells. Before that, this interconnection was only hinted at or analysed in *in vitro* data.

**What do you enjoy most about being an early-career researcher?**

I really enjoy the fact that, in the early stages of your career, you can really pursue your scientific curiosity.

**What piece of advice would you give to the next generation of researchers?**

Scientific progress is often made in incremental steps. Not everything can be groundbreaking and novel; even the little advances can contribute meaningfully to the field and help us to better understand our world.

**What's next for you?**

Currently, my main focus is to start my internship abroad and, once I am back, to finish my PhD. After that, we will see what awaits me.
